# The effect of turmeric and black pepper powder incorporated in breakfast on postprandial glycemia, appetite, palatability, and gastrointestinal well‐being in normal‐weight adults

**DOI:** 10.1002/fsn3.3965

**Published:** 2024-01-16

**Authors:** Safarat Khan, Muhammad Arif, Hafza Laraib, Syeda Nimra Naqvi, Omair Ali Shah, Umar Farooq, Muhammad Sami‐Ullah, Gul Asghar Khan

**Affiliations:** ^1^ Department of Human Nutrition The University of Agriculture Peshawar Peshawar Pakistan; ^2^ University Institute of Diet and Nutritional Sciences The University of Lahore Islamabad Pakistan; ^3^ Spinghar Institute of Higher Education Jalalabad Afghanistan

**Keywords:** appetite, black pepper, piperine, postprandial Glycemia, turmeric

## Abstract

Culinary herbs and spices are primarily known as flavor enhancers, research suggests that black pepper (*Piper nigrum*) and turmeric (*Curcuma longa*) have now been proven to prevent many non‐communicable chronic diseases such as diabetes. Bioactive components of black pepper and turmeric ameliorate glucose metabolism and appetite regulation. The present research was designed to investigate the impact of turmeric and black pepper on blood glycemia, gastrointestinal well‐being, appetite, and palatability. In a randomized crossover study, four iso‐caloric experimental meals each having 50 g of available carbohydrates were subjected to healthy human participants (*N* = 20). Turmeric and black pepper were incorporated in the breakfast meal, 1 g black pepper (BP), 1 g turmeric (TR), and combination of the (BP + TR) was added in the breakfast. Standard questionnaires were used to evaluate palatability, subjective appetite, and gastrointestinal well‐being. Blood glycemia, subjective gastrointestinal well‐being, and appetite were measured at 0, 30, 60, 120, and 180 min. Experimental meals BP and BP + TR resulted in lower blood glycemia (*p* < .05) significantly compared to control meal. A decrease in perceived eating ability and hunger, and an increase in satiety after BP + TR and BP meal was observed. No significant changes were observed after consuming test meals on gastrointestinal well‐being. Compared to control and BP + TR meals, BP and TR meals had considerably lower palatability. Results showed that compared to the control intake of starchy meals supplemented with black pepper and turmeric reduced postprandial glycemia, hunger, and perceived eating ability without affecting gastrointestinal well‐being.

## Graphical abstract

Meal supplemented with black pepper and turmeric reduced postprandial glycemia, hunger, and perceived eating ability without affecting gastrointestinal well‐being.

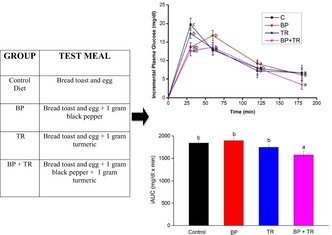

## 1 INTRODUCTION

Diabetes mellitus (DM) is a chronic disease resulted by pancreatic beta cell dysfunction and/or deterioration of glycemic control by other disorders. Chronic hyperglycemia through multiple mechanisms have deleterious consequences on insulin secretion/synthesis in addition to insulin sensitivity. Other factors including oxidative stress, gradual malfunctioning in gene expression of insulin and other beta‐cells, persistent endoplasmic reticulum strain, and morphological transformation of membranous mitochondria, also contribute to diabetes (Cernea & Dobreanu, 2013). DM is categorized by restricted or entire absence of insulin and interruption in carbohydrates, protein, and fat metabolism (American Diabetes Association, 2010). In developing countries, DM is a common public health issue and appeared as a huge socioeconomic load that touched epidemic proportions worldwide. In Pakistan, the first survey (1994–98) revealed an 8.7% prevalence. Globally, there were 451 million people with diabetes in 2017. A recent survey reported that about 26.3% of the Pakistani populace over 19 year's of age is diabetic (Ijaz et al., 2020).

There is a strong connection between the diet quality and DM. Postprandial glucose level is directly affected by the quantity and quality of starch in diet (Blaak et al., 2012; Punthakee et al., 2018). Drug and diet therapy have been widely used approaches and currently, the focus is on spices and natural herbs to treat DM. Hypoglycemic drugs are not only costly but have several unpleasant side effects such as severe hypoglycemia, abdominal discomfort, peripheral edema, and lactic acidosis (Ghorbani, 2013; Marín‐Peñalver et al., 2016), whereas bioactive compounds derived from natural resources are regarded as cost‐effective and safe (Rao & Jamil, 2011).

Many studies focused on bioactive properties indicated that food has a pivotal role in the prevention of chronic non‐communicable diseases and can be recommended for the treatment and management of DM (Bi et al., 2017; Jungbauer & Medjakovic, 2012; Kaefer & Milner, 2008; Tapsell et al., 2006; Wannes & Marzouk, 2016). Medical nutrition therapy (MNT) helps in the diminution of hypoglycemia risk, eliminating major symptoms and slowing down the long‐term microvascular complications associated with diabetes (Bantle et al., 2008).

A single herb has multiple actions on numerous ailments, also has synergistic and antagonist effects in combination with other herbs (Umashanker & Shruti, 2011). Black pepper (*Piper nigrum*) family piperaceae, with the main active ingredient piperine, has a hypoglycaemic effect (Panda & Kar, 2003). Turmeric (Curcuma longa), family Zingiberaceae, in which a vital active ingredient is curcumin, has been revealed to have hypoglycaemic, antioxidant, and lipid‐lowering effects in many investigational studies (Khaliq et al., 2015). Earlier studies revealed that both herbs work in synergy in lowering postprandial blood glucose levels. Previously such types of investigations were conducted only on animals not on humans. Therefore, the key objective of this study was to evaluate the impact of black pepper and turmeric separately and in combination on postprandial blood glycemia, gastrointestinal well‐being, appetite, and palatability. It was hypothesized that black pepper and turmeric together have a greater effect on lowering postprandial glycemia.

## 2 MATERIALS AND METHODS

The study was carried out in the laboratory of clinical dietetics and nutritional biochemistry at The University of Lahore (UOL), Islamabad Campus, Islamabad Pakistan.

### 2.1 Ethical consideration and consent

This Human study was approved by the Ethical Committee and Human Studies Review Board (FNS‐ECHSRB/2015–0091) of The University of Agriculture, Peshawar. The current research followed guidelines proposed in line with the guidelines proposed in the Helsinki Declaration. Informed written consent was obtained from the study participants.

### 2.2 Participants and screening procedure

For this study, a total of 20 healthy individuals comprising 10 males and 10 females, were enrolled through direct personal communication. Inclusion criteria were: free from chronic diseases, aged 18–60 years, and healthy individual with body mass index (BMI) (18.5–24.9 kg/m^2^) medically healthy. Exclusion of the subjects from the study was made on the following rationale: Dislike for egg, vegetarian, unwillingness to give blood via finger prick, smoking, restricted dietary habits, unusual weight alternation >10 pounds in the last 3 months, taking medications regularly, using antibiotics for the last 3 months, not normally consuming breakfast and not willing to participate in the study.

### 2.3 Study design and intervention

The study made use of a randomized crossover design; all the subjects were provided with 04 test foods, all have 50 g carbohydrate content. Every subject attended four sessions with 1 week washout period between the trials. The subjects were requested to consume one of the experimental meals on each day of assessment. Subjects were directed to abstain from any tiring physical exertion a day prior to their visit. With the intention of reducing the variation in the effect of subsequent meals, subjects were asked to maintain a similar dietary pattern the evening before each visit and to document this in a memoir. Selected personnel were instructed to take their twilight meal up to 10:00 p.m. following which they were asked to fast (consumption of water with an upper limit of 500 mL (half a liter) was permissible amid 10:00 p.m. and the next daybreak). Upon the influx of subjects into the research lab, baseline data involving height and body weight were deliberated. After a respite of 5 min, fasting capillary blood glucose concentration was measured by finger prick, and the testimony of the subject's intensity of appetite and hunger was reported in the course of visual analog scales (VAS) (time 0). Next, the test meals, supplying 50 g of accessible carbohydrates, were ingested in 10 min. In the interim, the subjects were obliged to gauge the food palatability via a 9‐score hedonic scale. Glucose concentration of blood was delineated at intervals of 30, 60, 120, and 180 min following the commencement of the food trial. The subjects were then solicited to fill up the VAS, right after the collection of each blood sample. Additionally, subjects were requisite to evaluate any gastrointestinal discomforts they observed, at fasting and at 30, 60, 120, and 180 min intervals after imbibing the food on trial. The ingestion of any kind of food or beverage during the period of 3 h was precluded with the exception of reading newspaper, watching small screen, or utilizing their workstation. It was acceptable for the study subjects to utter freely to each other provided that they do not speak of topics related to food and appetite.

### 2.4 Test meal

All the test meals were prepared in the laboratory of clinical dietetics and nutritional biochemistry of UOL Islamabad Campus, Islamabad Pakistan. The research study was comprised of four trial visits. Test foods were: (1) bread toast and egg (control diet), (2) bread toast and egg +1 g black pepper (BP), (3) bread toast and egg +1 g turmeric (TR), and (4) bread toast and egg +1 g black pepper +1 g turmeric (BP + TR), all meals were served with tea. All the food items including bread, egg, black pepper, turmeric, salt, oil, milk, sugar, and tea were bought from the market. The control breakfast was prepared by taking an egg mixed with 0.4 g salt and fried in 5 g oil for 1 min. In test meals black pepper (1 g) and turmeric powder (1 g) were added to the egg. The fried egg was served with 2 standard bread toasts and a cup of black tea (250 mL) having 200 mL milk, 50 mL water, 1 teaspoon black tea (5 g), and 1 tablespoon sugar (8 g). The ingredient composition (qualitative and quantitative) of the meal used in the trial is given in Table 1.

**TABLE 1:** Ingredients and nutrient composition.

Ingredients	Control	BP	TR	BP + TR
Bread (g)	90	90	90	90
Egg (g)	50	50	50	50
Oil (g)	10	10	10	10
Salt (g)	0.4	0.4	0.4	0.4
Black pepper (g)	–	1	–	1
Turmeric (g)	–	–	1	1
Water (mL)	200	200	200	200
Sugar (g)	08	08	08	10
Black tea (g)	5	5	5	5
Total weight	413.4	414.4	414.4	415.4
Energy (g)	581	581	581	581
Average CHO (g)	50	50	50	50
Proteins (g)	21	21	21	21
Fats (g)	24	24	24	24
Dietary fiber (g)	1.7	1.7	1.8	1.8

Abbreviations: BP, Black pepper; BP + TR, Black pepper and turmeric; CHO, Carbohydrates; TR, Turmeric.

### 2.5 Anthropometric and biochemical measurements

Body weight and height were assessed through standard methods. Weight was measured through a digital scale, the subjects were asked to remove heavy clothes, shoes, and other unnecessary things. The height was measured through a stadio‐meter. Body mass index was calculated according to WHO categorization as underweight, normal, overweight, or obese (World Health Organization, 1995).

Glucometer with specifications (Freestyle Optium; Abbott Diabetes Care, Inc., Berkshire, UK) was employed to compute the blood glucose, and samples were collected via finger prick. The values were recorded in the blood glucose record sheet (Wolever, 2004), blood glucose levels were recorded at 0, 30, 60, 120, and 180 min. Using a controlled elucidation from the producer, the quality control tests were executed in the morning of each trial day where the coefficient value was 0.6% of deviation of these quality control appraisals.

### 2.6 Appetite assessment

Appetite was assessed by using VAS (Flint et al., 2000). The VAS consisted of four scales, each of 100 mm length, which was used to determine the participant's hunger from 0 to 100 mm like (0 = not hungry,100 = very hungry), satisfaction(0 = not satisfied, 100 = very satisfied), fullness(0 = not full, 100 = very full), and prospective food intake (0 = nothing at all, 100 = a lot). Subjects were fully guided about to mark a vertical line on scales, between the 0 and 100 mm line. After that, these values were converted to continuous variables. At the time of marking scales, avoid referring the previous rating.

$$\text{Composite appetite score or}\;\mathrm{CAS}=\left[\text{satiety}+\text{fullness}+\left(100-\text{prospective food composition}\right)+\left(100-\text{hunger}\right)\right]/4$$

### 2.7 Palatability of the test foods

A 9‐score hedonic scale was exercised in this study for appearance, taste, texture, and overall acceptability for assessing the palatability of all four test meals. A 9‐point hedonic calibration was exploited with dislike extremely on one end and like extremely on the other end. Then subjects have their own choice to mark a position on the scale anywhere, that best matches their perception (Meilgaard et al., 2007).

### 2.8 Gastrointestinal well‐being

Gastrointestinal symptoms such as swelling of stomach, uneasy feelings, flatulence, loose stools, diarrhea, constipation, cramping, and grumbling were assessed using a 4‐point Likert scale. Responses were recorded at fasting (0 min) and time points 30, 60, 90, 120, and 180 min. The responses were rated as 1—none, 2—mild, 3—moderate, and 4—severe (Bonnema et al., 2010).

### 2.9 Calculation and statistical analysis

The sample size was calculated using NCSS 2004/PASS 2002 software (Hintz, 2001). SPSS version 16 was used for analyzing statistical data for examining the outcome of treatment, effect of time, and the relationship between time and treatment for evaluating glucose level, hunger, fullness, satiety, prospective food intake, and gastrointestinal well‐being. If there was a significant effect, then apply a one‐way repeated measurement (ANOVA). Then mean differences among treatments at each time point were explained with the help of Bonferroni adjustment for multiple comparisons. The effect on appetite parameters; hunger, fullness, satiety, prospective food intake, and blood glucose were examined by one‐way recurrent measurement (ANOVA) which was further tracked by post hoc check with Benferroni statistical tool for manifold comparisons to determine any significant effect among the test meals in the palatability parameters. Significances were indicated when (*p* < .05).

Incremental values of glucose level and appetite parameters for test meals were measured by subtracting fasting values from postprandial values. Then these values were used to construct the incremental response curves. In order to approximate the incremental locale beneath the arc for blood glucose, satiety, and fullness (ignoring area under zero), the trapezoidal rule was made use of. With the same method also calculate the incremental area over the curve for hunger and prospective food intake (ignoring the area above zero).

## 3 RESULTS

### 3.1 Demographic and anthropometric characteristics of studied subjects

The demographic and anthropometric attributes of the studied subjects are shown in Table 2. The mean age of female students was 21.4 ± 0.72 years with a mean BMI of 23.5 ± 3.9 and fasting blood glucose of 73.5 ± 9.05 mg/dL. For boys the mean age was 21.9 ± 0.84, BMI was 23.22 ± 5.7, and fasting blood glucose was 77.35 ± 9.57 mg/dL.

**TABLE 2:** Demographic and anthropometric characteristics of studied subjects (n = 20).

Variable	Mean ± SD	
	Female	Male
M/F	10	10
Age (y)	21.4 ± 0.72	21.9 ± 0.84
Weight (kg)	56.8 ± 6.75	68.5 ± 9.15
Height (cm)	155.2 ± 2.88	172.72 ± 11.4
Waist circumference (cm)	77.2 ± 6.78	86.48 ± 13.4
BMI (kg/m^2^)	23.5 ± 3.9	23.33 ± 5.7
Fasting plasma glucose (mg/dL)	73.5 ± 9.05	77.35 ± 9.57

Abbreviations: BMI, Body mass index; SD, Standard deviation.

### 3.2 Blood‐sugar response

**FIGURE 1:**
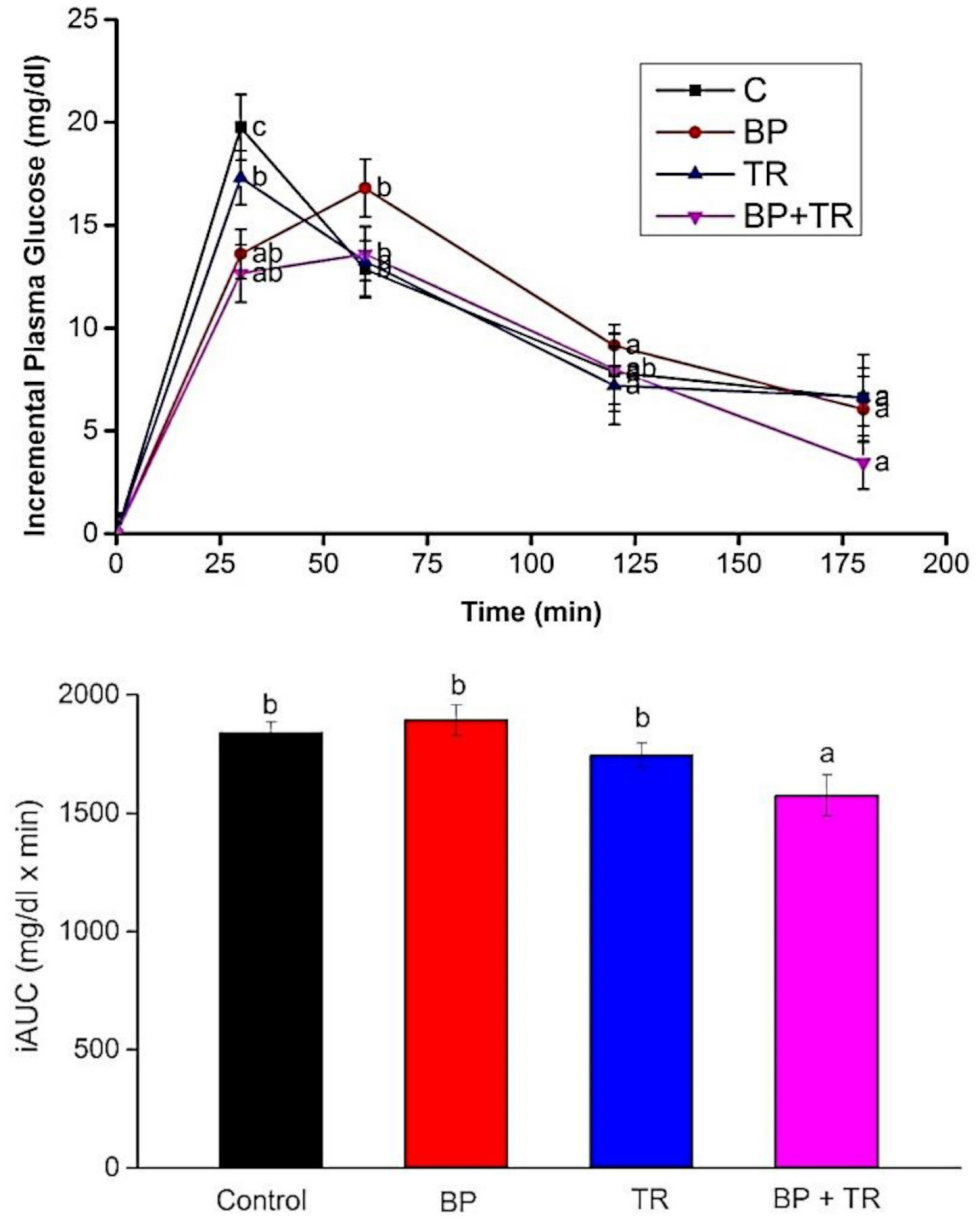
Mean (±SEM) deviates from the baseline in blood sugar and incremental area under the curves (iAUCs) in 20 healthy subjects after consuming the experimental meals. BP, black pepper; BP + TR, Black pepper and turmeric; C, control meal; TR, turmeric. Vertical bars having same letters are non‐significant, while having different letters are significant through one‐‘way repeated (ANOVA) (*p*‐value <.05).

Incremental area under the blood glucose response curves (iAUCs) after the consumption of test meals is shown in Figure 1. For blood glucose level the significant effect was shown by time (*p* < .001) and time × treatment (*p*‐value <.001) and treatment (*p*‐value <.001). Therefore, test meals (BP and BP + TR) show a significant effect for blood glucose level as compared to the reference meal (C) (*p* < .05) by Post hoc twosome comparison. Repeated one‐way (ANOVA) was applied to investigate the further ramification of time and time × treatment interaction. So test meals (BP and BP + TR) show a significant effect for blood glucose level at the time point 30 min as compared to reference meal (C) (*p* = .023). Incremental area under the curves (iAUCs) among the test meals shows a non‐significant effect.

### 3.3 Subjective appetite measure

**FIGURE 2:**
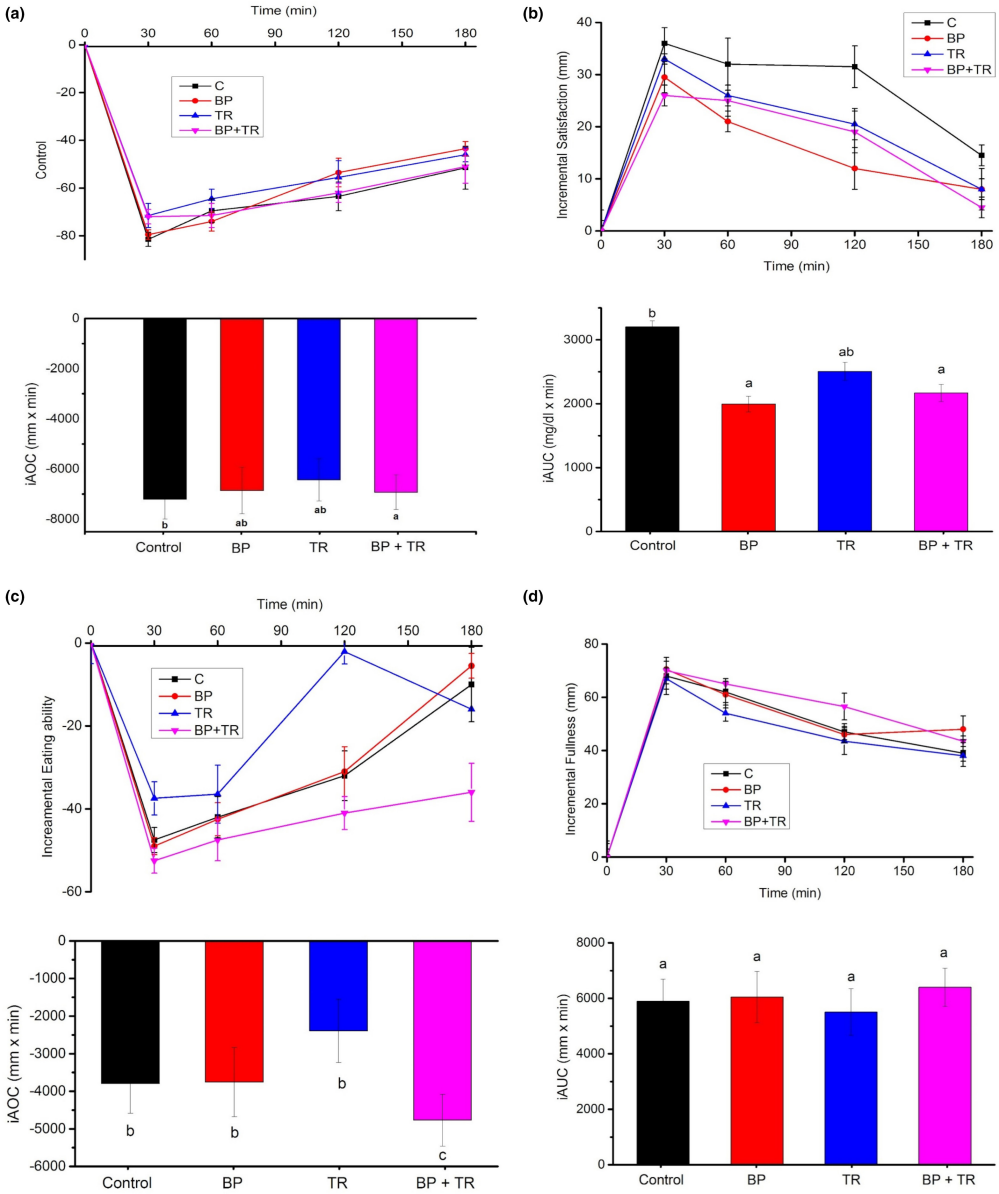
Mean deviates from the baseline in response to appetite parameters; (a) hunger, (b) satisfaction, (c) prospective eating ability, and (d) fullness with consequent incremental areas under or above the curves (iAUCs/iAOCs) of all the 20 healthy study subjects after consuming test meals. BP, black pepper; BP + TR, black pepper and turmeric meal; C, control meal; TR, turmeric. Vertical bars having same letters were not significantly (*p* > .05) different while having different alphabets were significant (*p*‐value <.05) through two‐way frequent measures (ANOVA).

Incremental area under or above the curves (iAUCs/iAOCs) for the limitations of appetite in terms of hunger, satisfaction, prospective eating ability, and fullness are shown in Figure 2a–d.

### 3.4 Palatability of all the four test meals

Table 3 depicts the Palatability statistics of all the four test meals. A significant difference (*p* < .05) was seen in the palatability parameters among the test meals. Appearance, texture, smell, taste, and overall pleasantness were significantly (*p* < .05) different among the test meals. Post hoc pair‐wise comparison shows that the control meal and combined meal have significantly (*p* < .05) higher acceptability than (BP and TR) meal in comparison of general appearance, consistency, smell, taste, and whole pleasantness.

**TABLE 3:** Palatability Statistics for all the Four Test Meals.

Test meal	Mean ± SD				
	Appearance	Texture	Smell	Taste	Overall pleasantness
C	6.3 ± 0.48^b^	6.4 ± 0.46^b^	5.5 ± 0.51^a^	6.7 ± 0.48^b^	6.5 ± 0.39^ab^
BP	5.9 ± 0.55^a^	5.35 ± 0.61^a^	6.05 ± 0.58^ab^	5.9 ± 0.55^a^	6.2 ± 0.50^a^
TR	5.7 ± 0.48^a^	5.5 ± 0.47^a^	5.6 ± 0.533^a^	5.9 ± 0.47^a^	6.1 ± 0.40^a^
BP + TR	6.5 ± 0.49^b^	6.2 ± 0.52^b^	6.2 ± 0.46^b^	6.5 ± 0.44^b^	6.6 ± 0.42^b^

*Note*: Means having same letters are not significantly (*p* > .05) different while having different alphabets are significantly different (*p*‐value <.05).

Abbreviations: BP + TR, Black pepper and turmeric; BP, Black pepper; C, control meal; SD, standard deviation; TR, Turmeric.

### 3.5 Gastrointestinal well‐being

**FIGURE 3:**
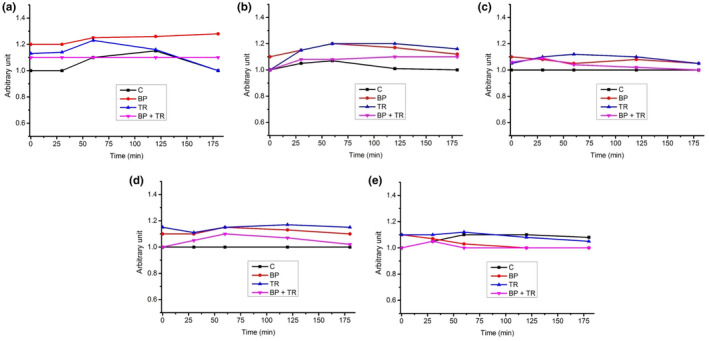
Mean responses of subjects of gastrointestinal symptoms; (a) gas or bloating, (b) nausea, (c) diarrhea, (d) constipation, and (e) abdominal pain. BP, black pepper; BP + TR, black pepper and turmeric meal; C, control meal; TR, turmeric. There was a non‐significant (*p* > .05) effect shown by these gastrointestinal symptoms, two‐way repeated measure (ANOVA).

The responses in rating of gastrointestinal symptoms are shown in Figure 3; A gas or bloating, B nausea, C diarrhea, D constipation, and E abdominal pain. There was no significant (*p* > .05) effect of time, treatment, and time × treatment on postprandial responses of gastrointestinal symptoms.

## 4 DISCUSSION

This research outlined the effect of black pepper and turmeric supplemented with breakfast on postprandial blood glycemia, gastrointestinal well‐being, palatability, and appetite among healthy individuals. It is hypothesized that supplemented breakfast would reduce glycemia without affecting palatability. This was the first scientific approach for testing the additive effects of black pepper and turmeric added to breakfast on blood glycemia in humans. The study results conclude that in contrast to other test meals, BP + TR meal markedly reduced postprandial blood glucose and test meals also have a significant impact on appetite without reporting any gastrointestinal discomfort. Breakfast meal was enjoyed by the study subjects; however, control meal and BP + TR meal have high palatability.

The observed decrease in postprandial blood glucose after taking BP and BP + TR is consistent with the study of Atal et al. (2012) who determined the bio‐enhancing result of metformin and black pepper in decreasing blood glucose in alloxan‐induced diabetic mice. These mice were split into four different groups. The black pepper and metformin together induce a greater reduction in plasma glucose concentration than metformin alone. Black pepper has the potential to be used as a bio‐enhancing agent in grouping with metformin which supports to lessen the amount of metformin and its other side effects.

The mechanism behind how experimental meals BP and BP + TR lower blood glycemia can be understood from these previous studies. Pepper active component piperine has been found to increase the bioavailability of turmeric by twofolds (Arcaro et al., 2014). Adding black pepper in combination with turmeric can increase bioavailability (Upasani et al., 2013). Another study conducted on normal mice also showed the hypoglycemic effect of piperine (Panda & Kar, 2003).

Contradictory to the assumption of this current approach, no remarkable effect of TR (1 g) meal on postprandial blood sugar level was noticed. The current result was in concurrence with the findings of Amin et al. (2015), their study results showed that turmeric alone (2.4 g/day), had no effect on blood glucose in males. Experimental meal TR did not ameliorate postprandial glycemia in contrast to the control meal. A possible rationale for this may be the use of low dose of turmeric powder in this study. Wickenberg et al. (2010) investigated the impact of turmeric on plasma glucose and insulin following a meal intake in normal human beings. They evaluated that the utilization of 6 g turmeric resulted in improved insulin levels while decreasing plasma glucose. However, in the current study, test meal supplemented with 1 g turmeric powder did not reduce the blood glycaemia which can be justified on the basis of low dosage. The previous animal and human studies plan was dissimilar from the current investigation. Further studies are required to find out the effect of turmeric with increased doses on glycemia with sustained palatability of the experimental meals.

In the present study, test meals have a significant effect on parameters of appetite, except fullness. BP and BP + TR compared to TR meal remarkably reduced perception of hunger followed by control. Control meal has high satisfaction followed by BP + TR meal, BP meal and TR meal, the increased satisfaction in present study is in concurrence with the results of Zanzer et al. (2019), their study results depicted that control meal has high perceived satiety and turmeric consumption prior to control did not modulate the perceived satiety. They further concluded that under their meal challenge conditions, dose of turmeric might not have been sufficient to elicit appetite‐associated effects as in our research. However, there is no reported study that has investigated the synergistic impact of black pepper and turmeric on appetite in humans. The decrease in hunger and perceived eating ability and increase in satiety and fullness after BP + TR and BP meal in this acute intervention are in agreement with the results of Zanzer et al. (2018), who observed appetite regulation of normal individuals after consumption of black pepper based drink. According to their study, the test meal significantly decreased hunger and perceived eating ability while increasing satiety and fullness.

However, in our study, the effect of treatment on fullness is not statistically significant but both of these meals (BP and BP + TR) have increased fullness as compared to TR and control meal. Several mechanisms are there to clarify the greater effect of black pepper on satiety and lowering glucose level. It was found that these effects are owing to the bio‐active profile of black pepper, such as isopiperine, isochavicine, piperine, piperanine, piperolein A and B, which might act on receptor TRPV1; transient receptor potential cation channel subfamily V member 1 (McNamara et al., 2005; Okumura et al., 2010). TRPV1 is present in the oral cavity and its activation is widely known to improve glucose homeostasis, potentially affect appetite responses as well as increase energy metabolism. Furthermore, TRPV1 activation increases GLP‐1; glucagon‐like peptide‐1 levels in the ileum and plasma. GLP‐1 secretion stimulated by TRPV1 activation could be a promising approach for diabetes intervention (Luo et al., 2012; Wang et al., 2012). However, hormones involved in the regulation of satiety were not examined in this intervention. In this research, all the test meals were acceptable however, a decrease in palatability of TR followed by BP, BP + TR in contrast to C was observed. The reported reduction in hunger after BP and BP + TR meal consumption might be because of decreased palatability since different scientific approaches have revealed an enormous effect of palatability on appetite (Sørensen et al., 2003).

This study has some deficiencies. The glycemic index of the test meals was not noted and it necessitates the need to be explored in future research studies. Besides, further future scientific research studies should be conducted to find a significant role of these spices including the role in and hormonal mechanisms for appetite regulation and mechanism gastric emptying. Further scientific investigations are required for evaluating the effect of turmeric and black pepper‐based products effect on blood cholesterol and other diseases.

## 5 CONCLUSION

The current interventional study depicts information on glycemic effects of breakfast supplemented with turmeric and black pepper in combination. The breakfast supplemented with BP and TR significantly decreased postprandial glycemia, hunger, perceived eating ability, and increased satiety in healthy individuals. All test meals were pleasant without effecting gastrointestinal health. The potential components of turmeric and black pepper and actual physiological mechanisms involved in these effects need further investigation. Future long‐term studies with increased dosage of turmeric and black pepper are considered necessary to understand the advantages of long‐term utilization of turmeric and black pepper supplemented breakfast in alleviating the risk of developing chronic diseases.

## AUTHOR CONTRIBUTIONS

**Safarat Khan:** Data curation (equal); formal analysis (equal); investigation (lead); methodology (lead); project administration (lead); writing – original draft (equal). **Muhammad Arif:** Data curation (equal); methodology (equal); supervision (lead); writing – original draft (equal). **Hafza Laraib:** Investigation (equal); methodology (equal); project administration (equal); writing – review and editing (equal). **Syeda Nimra Naqvi:** Investigation (equal); methodology (equal); project administration (equal); writing – review and editing (equal). **Omair Ali Shah:** Investigation (equal); methodology (equal); project administration (equal). **Umar Farooq:** Formal analysis (equal); investigation (equal); project administration (equal); supervision (equal); writing – original draft (equal). **Muhammad Sami‐Ullah:** Data curation (equal); formal analysis (equal); supervision (equal); writing – original draft (equal). **Gul Asghar Khan:** Investigation (equal); project administration (equal); writing – review and editing (equal).

## CONFLICT OF INTEREST STATEMENT

No conflict of interest.

## Data Availability

The data that support the findings of this study are available on request from the corresponding author.
